# Meldonium, as a potential neuroprotective agent, promotes neuronal survival by protecting mitochondria in cerebral ischemia–reperfusion injury

**DOI:** 10.1186/s12967-024-05222-7

**Published:** 2024-08-15

**Authors:** Weijie Yang, Xiuxing Lei, Fengying Liu, Xin Sui, Yi Yang, Zhenyu Xiao, Ziqi cui, Yangyang Sun, Jun Yang, Xinyi Yang, Xueyang Lin, Zhenghao Bao, Weidong Li, Yingkai Ma, Yongan Wang, Yuan Luo

**Affiliations:** 1 State Key Laboratory of Toxicology and Medical Countermeasures, Beijing Institute of Pharmacology and Toxicology, Beijing, China; 2 Lu’An Hospital of Traditional Chinese Medicine, Anhui, China

**Keywords:** Meldonium, Cerebral ischemia–reperfusion injury, Neurons, Mitochondria

## Abstract

**Background:**

Stroke is a globally dangerous disease capable of causing irreversible neuronal damage with limited therapeutic options. Meldonium, an inhibitor of carnitine-dependent metabolism, is considered an anti-ischemic drug. However, the mechanisms through which meldonium improves ischemic injury and its potential to protect neurons remain largely unknown.

**Methods:**

A rat model with middle cerebral artery occlusion (MCAO) was used to investigate meldonium’s neuroprotective efficacy in vivo. Infarct volume, neurological deficit score, histopathology, neuronal apoptosis, motor function, morphological alteration and antioxidant capacity were explored via 2,3,5-Triphenyltetrazolium chloride staining, Longa scoring method, hematoxylin and eosin staining, terminal deoxynucleotidyl transferase-mediated dUTP-biotin nick end labeling assay, rotarod test, transmission electron microscopy and Oxidative stress index related kit. A primary rat hippocampal neuron model subjected to oxygen–glucose deprivation reperfusion was used to study meldonium’s protective ability in vitro. Neuronal viability, mitochondrial membrane potential, mitochondrial morphology, respiratory function, ATP production, and its potential mechanism were assayed by MTT cell proliferation and cytotoxicity assay kit, cell-permeant MitoTracker^®^ probes, mitochondrial stress, real-time ATP rate and western blotting.

**Results:**

Meldonium markedly reduced the infarct size, improved neurological function and motor ability, and inhibited neuronal apoptosis in vivo. Meldonium enhanced the morphology, antioxidant capacity, and ATP production of mitochondria and inhibited the opening of the mitochondrial permeability transition pore in the cerebral cortex and hippocampus during cerebral ischemia–reperfusion injury (CIRI) in rats. Additionally, meldonium improved the damaged fusion process and respiratory function of neuronal mitochondria in vitro. Further investigation revealed that meldonium activated the Akt/GSK-3β signaling pathway to inhibit mitochondria-dependent neuronal apoptosis.

**Conclusion:**

Our study demonstrated that meldonium shows a neuroprotective function during CIRI by preserving the mitochondrial function, thus prevented neurons from apoptosis.

## Introduction

Stroke rates have risen among young people due to irregular daily routines and overeating. Stroke is the second leading cause of death, accounting for approximately 11% of global fatalities [1]. Ischemic stroke comprises approximately 74% and 85% of stroke cases in China and the United States respectively. This disease exhibits a high prevalence, with limited therapeutic interventions available [2, 3]. Blood clots in cerebral arteries reduce or stop blood flow in an ischemic stroke. Clots usually resolve within several minutes to hours, restoring blood flow. Neurons die quickly by necrosis in ischemic cores, which solely depend on blocked arteries for blood. The ischemic penumbra, adjacent to the damaged area but dependent on other arteries for blood supply, undergoes delayed apoptosis [4]. Neuronal impairment reduces the quality of life even in stroke survivors. However, tissue plasminogen activator (tPA) remains the only effective drug approved by the United States Food and Drug Administration (FDA) for acute ischemic stroke while the narrow time window restricts its application to only a limited number of stroke patients [5, 6].

Mitochondrial dysfunction is an indicator of neuronal death following an ischemic stroke [5]. Therefore, to maintain neuronal mitochondrial function in cerebral ischemia–reperfusion injury (CIRI) has emerged as a promising treatment strategy [7]. In CIRI, the absence of necessary glucose and oxygen disturbs the cerebral energy balance, leading to detrimental processes such as inflammatory response, oxidative stress, necrosis, apoptosis, and other pathologic phenomena [8, 9]. During reperfusion, restored blood flow also induces excessive production of reactive oxygen species (ROS), severely disrupting mitochondrial homeostasis and function [7]. Therefore, it is necessary to identify mitochondria-targeting drugs for CIRI treatment.

Meldonium (3-(2,2,2-trimethylhydrazinium) propionate; MET-88; quaterine) has been widely reported as an inhibitor of carnitine biosynthesis, and its primary role in the mitochondria allows it to modulate cellular energy metabolism [10]. Meldonium partially inhibits fatty acid oxidation and stimulates glucose use [11]. This pharmacological mechanism makes it effective for treating cardiac and renal acute ischemia–reperfusion injury [12–16]. Additionally, meldonium has shown potential for treating stroke, convulsions, traumatic brain injury, and Parkinson’s disease [17–21]. A randomized, double-blind, multicenter clinical research demonstrated that meldonium injection is effective in treating acute cerebral infarction (ACI) [22]. However, despite many studies on treating neurological diseases with meldonium, the precise mechanism for treating CIRI remains unclear. Some reports indicate that cholinergic and glutamatergic pathways are involved in this mechanism [20], while others reveal a NO-dependent mechanism related to inducible nitric oxide synthase (iNOS) [23–25]. Since the brain does not use fatty acids as a fuel source, which indicates the mechanism of meldonium on the central nervous system (CNS) may be associated with an alternative carnitine-independent mechanism of action [26]. Considering that meldonium primarily impacts glycolysis and metabolic pathways [27], its mechanism of action in treating CIRI may involve promoting glucose use in the brain [10, 17, 26, 28].

In this study, we investigated the protective effects of meldonium on neurological deficits, infarct size, pathological lesions, and apoptosis in rats following middle cerebral artery occlusion (MCAO). Our findings revealed that CIRI causes severe oxidative stress in the brain. The inability to manage this stress would degrade mitochondrial structure and function, causing neuronal apoptosis, neurologic deficits, and cerebral infarction [29, 30]. However, we concluded that meldonium could alleviate these injuries. Furthermore, rat primary hippocampal neurons subjected to oxygen–glucose deprivation/reperfusion (OGD/R) were used to investigate the protective mechanism of meldonium on mitochondria. It has been found that meldonium primarily exerts its therapeutic effects on OGD/R neurons through the activation of the Akt/GSK-3β signaling pathway, therefore inhibiting the mitochondria-dependent neuronal apoptosis [31, 32].

## Materials and methods

### Animals and treatment

Male Sprague–Dawley rats weighing 220–240 g were purchased from Beijing Vital River Laboratory Animal Technology Co., Ltd. They were housed with a standard laboratory diet at least 7 days before the experiment at room temperature (22 ± 1 °C) with a 12 h light/dark cycle). All experimental procedures and treatments were approved by the Institutional Animal Care and Use Committee (IACUC number: IACUC-2012-010; National Beijing Center for Drug Safety Evaluation and Research, Beijing, China).

Animals were randomly categorized into six groups: (1) sham-operated rats (n = 8); (2) saline-treated MCAO rats (vehicle; n = 8); (3–5) MCAO rats that received meldonium at a dose of 50 mg/kg (n = 8), 100 mg/kg (n = 8) or 200 mg/kg (n = 8) (Liaoning Green Biological Pharmaceutical Group Co., Ltd., HPLC > 99%) [33]; and (6) MCAO rats that received edaravone at a dose of 10 mg/kg (Jiangsu Simcere Pharmaceutical Co., Ltd.) [34], as a positive control drug. Edaravone (3-methyl-1-phenyl-2-pyrazolin-5-one) is a fat-soluble free-radical scavenger that can significantly improves functional outcome in patients with acute ischemic stroke [35–39]. Therefore, we selected edaravone as the positive control. Meldonium, saline, and edaravone were administered intravenously 1 h after ischemia and 5 h after reperfusion.

### Establishment of MCAO-induced rat cerebral ischemic injury models

Rats underwent a transient 120 min filament occlusion of the right MCAO to induce ischemic stroke [17, 35]. 2% pentobarbital sodium (50 mg/kg, intraperitoneally) was used to anesthetize the rats. The right common carotid artery (CCA) was exposed by madding a midline neck incision. The right middle cerebral artery (MCA) was then occluded by a poly l-lysine thread 4–0 monofilament nylon (Beijing Cinontect, Beijing, China) 19 mm from the CCA. The filament was removed to allow brain tissue reperfusion 2 h later. The filament of sham-operated animals was not advanced into the MCA. After being returned to their cages, animals were reoffered water and food.

### Neurological impairment scores and infarct volume

The Longa scoring method was used to assess neurological impairment scores (n = 10) by a blinded investigator who scored 24 h after MCAO [40]. Additionally, the infarct volume was measured by 2,3,5-Triphenyltetrazolium chloride (TTC, Sinopharm Chemical Reagent, China) staining at 24 h after reperfusion (n = 5). Brain tissues were isolated and frozen at −20 °C for 20 min. Serially cutting the brains into 2 mm thick sections. Sections were immersed in 2% TTC for 30 min at 37 °C. The infarct volume was quantified using a digital camera and image analysis software (Motic, China). The infarct volume was calculated as the infarcted area/total non-infarcted ipsilateral hemisphere area.

### Hematoxylin and eosin (HE) staining

Rats (n = 5) were perfused through the heart by pre-chilling with 0.9% saline and 4% paraformaldehyde after reperfusion. The brains were placed in 10% formalin for over 24 h. Paraffin was embedded in the sections, followed by deparaffinization with xylene and descending grades of alcohol in water. Hematoxylin was used to stain the sections for 5 min, followed by eosin for 2 min.

### Terminal deoxynucleotidyl transferase-mediated dUTP nick end-labeling (TUNEL) staining

A TUNEL kit (Roche, Germany) was used to assess apoptotic cells following the manufacturer’s instructions. In brief, the sections of the brain were immersed in proteinase K at 37 °C for 30 min. Subsequently, the broken-membrane working fluid was added, and the cells were incubated at RT for 20 min. Next, reagents 1 (TdT) and 2 (dUTP) were used to incubate the sections for 2 h at 37 °C. After inactivating endogenous peroxidase by 3% H_2_O_2_ for 15 min, the sections were incubated in converter-POD at 37 °C for 30 min, added freshly DAB (DAKO, Denmark), and counterstained with hematoxylin. Finally, the ImageJ software was used to count apoptotic cells.

### Rotarod test

Three days before MCAO, all rats underwent pre-training on the rotating rod (3 times a day for 3 consecutive days). During this period, the rotating speed was adjusted from 4 to 40 rpm gradually, and then maintained 5 min. On the 3rd, 7th, and 14th day after reperfusion, a rotating rod test was conducted. The walking duration (s) of rats on the rotating rod was measured by the time data acquisition system.

### Ultrastructure observations

The cortex and hippocampus of rats (n = 3) were immediately isolated from the infarct penumbra (approximately 1 mm^3^) and placed in glutaraldehyde 24 h after reperfusion. Subsequently, the samples were dehydrated in a gradient, replaced with epoxy resin, embedded in paraffin, and sectioned into ultrathin sections. Changes in mitochondrial ultrastructure were observed using transmission electron microscopy (H-7650, AMT and HT7800/HT7700, hitachi, Japan).

### Measurement of malondialdehyde (MDA), glutathione (GSH) levels, and superoxide dismutase (SOD), total antioxidant capacity (T-AOC) activity

Rats (n = 10) were decapitated at 24 h after reperfusion, and the cortex and hippocampus of the infarct penumbra were isolated to determine the levels of malondialdehyde (MDA), glutathione (GSH), and superoxide dismutase (SOD), as well as the antioxidant capacity (T-AOC). Samples were properly treated, and the protein concentration was assayed using a BCA Protein Assay Kit (KeyGen Biological, China). T-AOC of the tissues, total SOD activity, GSH levels, and MDA levels were assayed using a T-AOC Assay kit, T-SOD Assay kit, GSH Assay kit and MDA Assay kit according to the manufacturer's instructions, respectively. All these kits were purchased from Nanjing Jianchen Biological Institute, China.

### Measurement of mitochondrial permeability transition pore (mPTP) opening and ATP level

The cerebral cortex and hippocampus of the infarct penumbra of the rats (n = 10) were isolated 24 h after MCAO. Mitochondria were isolated using a mitochondrial extraction kit (Nanjing Jianchen Biological Institute). After quantifying the mitochondrial protein concentration to 0.5 µg/µl using respiration buffer, mitochondrial permeability transition pore (mPTP) opening was induced by the addition of CaCl_2_ [41]. The absorbance was measured at 520 nm for 10 min using a spectrophotometer. The ΔA (A_max_–A_min_) indicated the extent of mPTP opening. The cortex and hippocampus in the infarct penumbra were isolated to detect ATP levels, which were assayed using an ATP assay kit (Nanjing Jianchen Biological Institute, Nanjing, China) based on phosphomolybdic acid colorimetry at a detection wavelength of 636 nm.

### Primary hippocampal neuron culture

Poly-d-lysine was used to coat 96/24/6-well plates 4 h in advance. The hippocampus of E17-E18 Wistar rat embryos was extracted and stored in a Ca^2+^ and Mg^2+^ free Hank's Balanced Salt Solution. The hippocampus was treated with trypsin (0.25%, 15 min, 37 °C) for 15 min and subsequently washed with Dulbecco’s Modified Eagle Medium (DMEM) containing 10% fetal bovine serum to obtain the cells of interest. The cells were plated at a density of (4.0–6.0) × 10^5^ cells/mL. The cells were maintained in a humidified incubator with 5% CO_2_ at 37 °C. After 4 h, the cells were washed with phosphate buffer saline (PBS) and the neurobasal medium supplemented with B27 (1:50) and GlutaMAXTM-1 (1:100) was used to replace DMEM. Primary hippocampal neurons were available for experiments after culturing for 7 days.

### Oxygen–glucose deprivation/reperfusion (OGD/R) model and drug treatment

For the OGD treatment, on day 7 of the culture, the neurobasal medium was replaced with glucose-free DMEM. Subsequently, the cells were placed in an anoxic incubator (Thermo, Waltham, MA) with mixed gas containing 1% O_2_, 5% CO_2_, and 94% N_2_ for 1 h. For reperfusion, glucose-free DMEM was replaced with neurobasal medium and incubated with 5% CO_2_ at 37 °C for the proper time.

### Assessment of cell viability

The 3-[4,5-dimethylthiazol-2-yl]-2,5-diphenyltetrazolium bromide (MTT) cell proliferation and cytotoxicity assay kit (Beyotime Biotechnology, China) was employed to test cell viability. In each culture well, 10 μL of MTT solution was added and incubated for 4 h at 37 °C. Subsequently, 100 μL of formazan solution was added and incubated for 4 h at 37 °C. Optical density values were recorded at a wavelength of 570 nm.

### Immunofluorescence assay

The cells were fixed in 4% paraformaldehyde (Electron Microscopy Services, 15,710) in 1 × PBS for 30 min at RT, washed, and incubated with 0.2% Triton. Subsequently, they were blocked with a blocking buffer (5% BSA, Fischer, BP1600). The cell-permeant MitoTracker® probes were incubated with the cells for over 12 h at 4 °C. Samples were imaged using high-content screening (Image Xpress Micro Confocal, Molecular Devices, Sunnyvale, CA, USA).

### Bioenergetic flux measurement by Seahorse XFe96

Real-time ATP rate (Cat. No.: 103592-100) and mitochondrial stress (Cat. No.: 103015-100) assays were conducted using a Seahorse XF HS Mini Analyzer (Agilent, Santa Clara, CA) to measure the proton efflux rate (PER) and oxygen consumption rate (OCR). The cells were plated at 2 × 10^5^ cells/well in XFp plates (Cat. No.: 103025-100). After OGD/R, Agilent Seahorse XF DMEM (Cat. No.: 103575-100) replaced the culture medium, supplemented with sodium pyruvate (1 mM), glutamine (2 mM), and D-glucose (1 mM). A 37 °C incubator without CO_2_ was used to keep cells at for 1 h. After using seahorse buffer to wash the cells, oligomycin (1.5 μM) and rotenone/antimycin A (Rot/AA) (0.5 μM) were prepared for measuring the real-time ATP rate, and oligomycin (1.5 μM), FCCP (1.0 μM), and Rot/AA (0.5 μM) were prepared for measuring OCR. Cells for mito-stress test were stained using 4',6-diamidino-2-phenylindole (DAPI) and counted using the fully automated cell imaging and analysis system (Falcon S300, Alicelligent Technologies, China). All experimental procedures were conducted at 37 °C. XF Wave Software (Seahorse Bioscience, Agilent) was used to analyze the data.

### Cellular reactive oxygen species (ROS) generation analysis

The cellular ROS formation was assessed using a DCFDA/H2DCFDA Cellular ROS Assay Kit (ab113851, Abcam, USA). Cells were cultured in 96-well plate and used for OGD/R experiment, after that ROS were detected according to the manufacturer’s protocol and measured by SpectraMax i/d5 (Molecular Devices, USA) at 485/532 nm.

### 5',6,6'-tetrachloro-1,1',3,3'-tetraethylbenzimidazolyl-carbocyanine iodide (JC-1) staining

The JC-1 (Dojindo Molecular Technologies, Tokyo, Japan) was used to assess mitochondrial membrane potential according to the manufacturer’s protocol. Briefly, cells were plated in a 24-well glass-bottom plate (Cellvis, USA) at a seeding density of 4 × 10^5^ cells/well. After the OGD/R injury, 2.0 μM JC-1 was added to react with the cells for 30 min. Subsequently, 100 μM of imaging buffer was added for imaging. All images were acquired and analyzed using high-content screening.

### Target screening for stroke through bioinformatics analysis

Search the online Comparative Toxicogenomics Database (https://ctdbase.org/), DrugBank (https://go.drugbank.com/), Therapeutic Target Database (TTD) (https://db.idrblab.net/ttd/), PharmGKB (https://www.pharmgkb.org/), Online Mendelian Inheritance in Man (OMIM) (https://www.omim.org/), and GeneCards (https://www.genecards.org/) for targets related to stroke by entering the word “stroke”. The results of the six database searches were combined to identify stroke targets. Org.Hs.eg.db was used for annotating the disease-related gene symbols. The Kyoto Encyclopedia of Genes and Genomes (KEGG) and Gene Ontology (GO) enrichment analyses of genes were conducted using ClusterProfiler and visualized with the ggplot2 mapping package. The PI3K-Akt pathway-related genes were analyzed using PathView. Protein–protein interaction (PPI) networks were analyzed with the (STRING) online database (http://string-db.org, Version 12.0). Cytoscape (version 3.8.0) was employed to identify gene intersections. Betweenness, closeness, degree, eigenvector, and network were used as screening criteria to identify 118 key genes to map the interaction network.

### Western blotting

Total protein of primary hippocampal neuronal cells was extracted by RIPA lysis and extraction buffer (Thermo Fisher Scientific, USA) following the manufacturer’s instructions. After preparing the sample, proteins were separated using 10% SDS-PAGE gel electrophoresis and semi-dry transferred to polyvinylidene fluoride (PVDF) membrane (Millipore, USA). Membranes were blocked with 5% BSA in Tris-buffered saline with 1% Tween 20 (TBST). After a 2 h incubation at RT, the membranes were left to incubate overnight at 4 °C with the following primary antibodies: Phospho-DRP1 (Ser637) Rabbit monoclonal antibody (mAb) (1:1000, Cell Signaling Technology, USA), Anti-Mitofusin 2 Mouse mAb (1:1000, abcam, UK), Anti-Mitofusin 1 Rbbit mAb (1:1000, abcam, UK), Anti-OPA1 Rbbit pAb (1:1000, abcam, UK), Akt rabbit mAb (1:1000, Cell Signaling Technology, USA), Phospho-Akt Rabbit mAb (1:1000, Cell Signaling Technology, USA), GSK-3β rabbit mAb (1:1000,Cell Signaling Technology, USA), Phospho-GSK-3β Rabbit mAb (1:1000,Cell Signaling Technology, USA), Anti-Cyclophilin D Mouse mAb (1:1000, Millopore, USA), Caspase-3 Rabbit mAb (1:1000, Cell Signaling Technology, USA), Cleaved Caspase-3 mAb (1:1000, Cell Signaling Technology, USA), Cytochrome c Rabbit mAb (1:500, Cell Signaling Technology, USA) and Actin Rabbit mAb (1:2000, abcam, UK). After washing with TBST, blots were incubated with horseradish peroxidase-conjugated secondary antibody (1:10000, ZSGB-BIO, Beijing, China) for 1 h at 35 ± 2 °C. Gray values of protein bands were quantified using Image Lab software (Bio-Rad, California, USA). The expression ratio of target proteins was normalized to the expression of β-actin.

### Statistical analysis

Statistical analyses were performed using GraphPad Prism (version 9.0, San Diego, CA, USA). All experimental values were expressed as mean ± standard deviation (SD). Comparisons between multiple groups were performed using one-way analysis of variance, followed by Tukey’s or Dunnett’s multiple comparison test. A significance level of *p* < 0.05 was considered statistically significant.

## Results

### Meldonium improves motor function and alleviates apoptosis in the cerebral cortex and hippocampus of MCAO rats

**Fig. 1:**
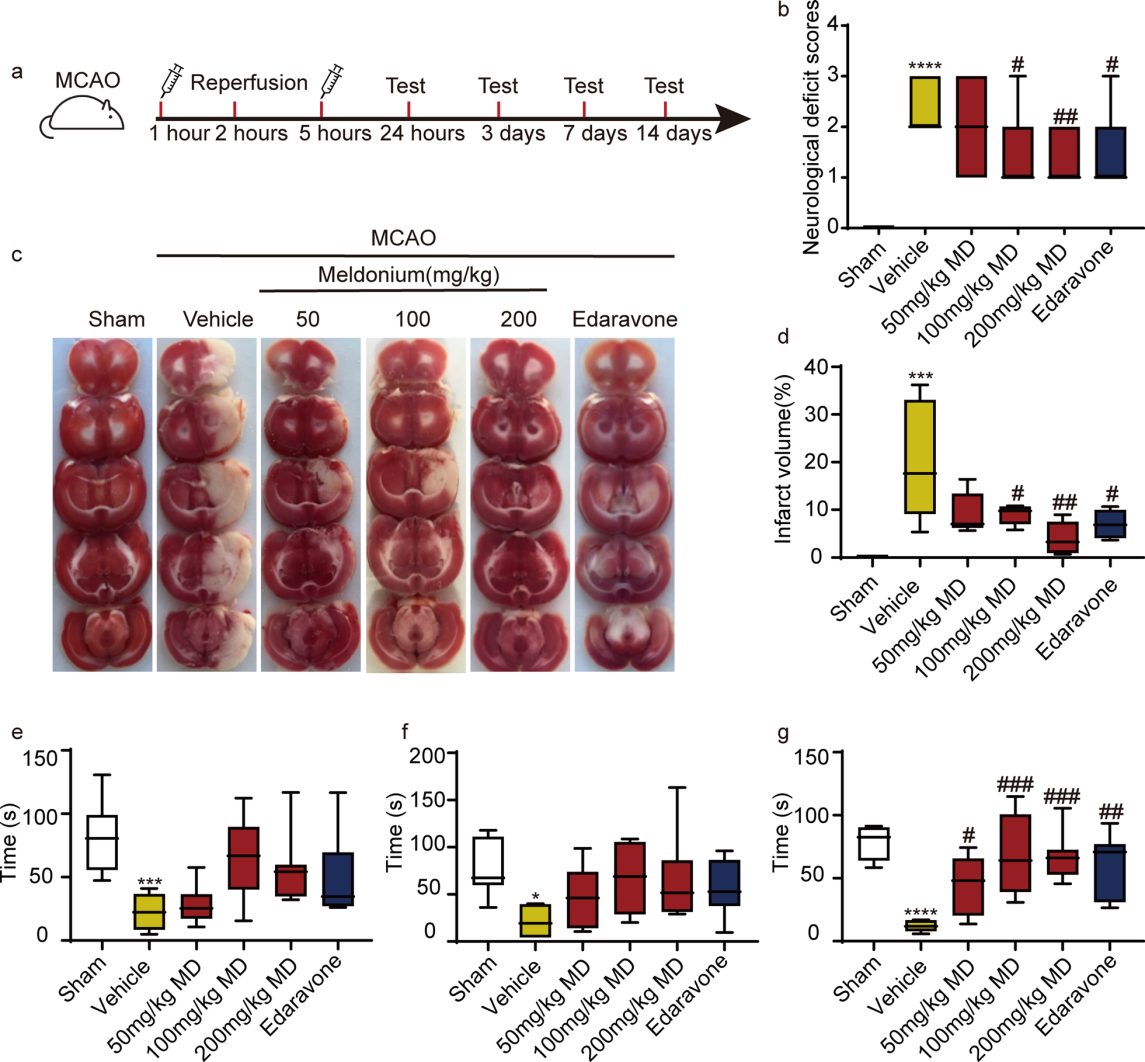
Meldonium treatment attenuates cerebral infarction and motor dysfunction in MCAO rats. **a** Diagram showing drug administration at specified times in the MCAO and reperfusion model. **b** Quantification of the neurological deficit score after reperfusion for 24 h. ****p < 0.0001 *vs*. the sham group; ^#^*p* < 0.05, ^##^*p* < 0.01 *vs.* the vehicle group. **c**, **d** Meldonium (50, 100, or 200 mg/kg) and edaravone reduced brain infarct size after 24 h of reperfusion; values are expressed as mean ± SD (n ≥ 8). ***p < 0.001 *vs*. the sham group; ^#^*p* < 0.05, ^##^*p* < 0.01, *vs*. the vehicle group.** e**–**g** Meldonium (50, 100, or 200 mg/kg) or edaravone treatment improved motor function in rats subjected to MCAO after reperfusion for 3 days (**e**), 7 days (**f**), and 14 days (**g**) as evaluated by the rotarod test. Values are expressed as the mean ± SD. (n ≥ 8). *p < 0.05, ***p < 0.001, ****p < 0.0001, *vs.* sham group; ^#^*p* < 0.05, ^##^*p* < 0.01, ^###^*p* < 0.001, *vs.* the vehicle group

MCAO-induced focal cerebral ischemia in rats subjected to 2 h of occlusion and 24 h of reperfusion was used to investigate the protective effect of meldonium against CIRI in vivo (Fig. 1a). Neurological deficits in the rats were determined using the Zea-Longa scoring criteria. The neurological deficit score (NDS) was significantly higher in MCAO rats compared to the sham-operated rats (Fig. 1b). However, treatment with 100 mg/kg, 200 mg/kg of meldonium, and edaravone significantly decreased NDS compared to the MCAO rats (Fig. 1b). TTC staining revealed a significant increase in cerebral infarction volume in MCAO rats (Fig. 1c, d), while treatment with 100 mg/kg and 200 mg/kg of meldonium and edaravone significantly reduced cerebral infarction volume compared to the MCAO rats (Fig. 1c, d). Furthermore, the rotarod test was used to evaluate motor function. The results revealed a significant decrease in the retention time on the rotating rod on the 3rd, 7th, and 14th days after MCAO (Fig. 1e–g). However, treatment with meldonium (Fig. 1g) and edaravone (Fig. 1g) increased the retention time on the rotating rod on the 14th day compared to the MCAO group, indicating that meldonium can improve the motor function of MCAO rats.

**Fig. 2:**
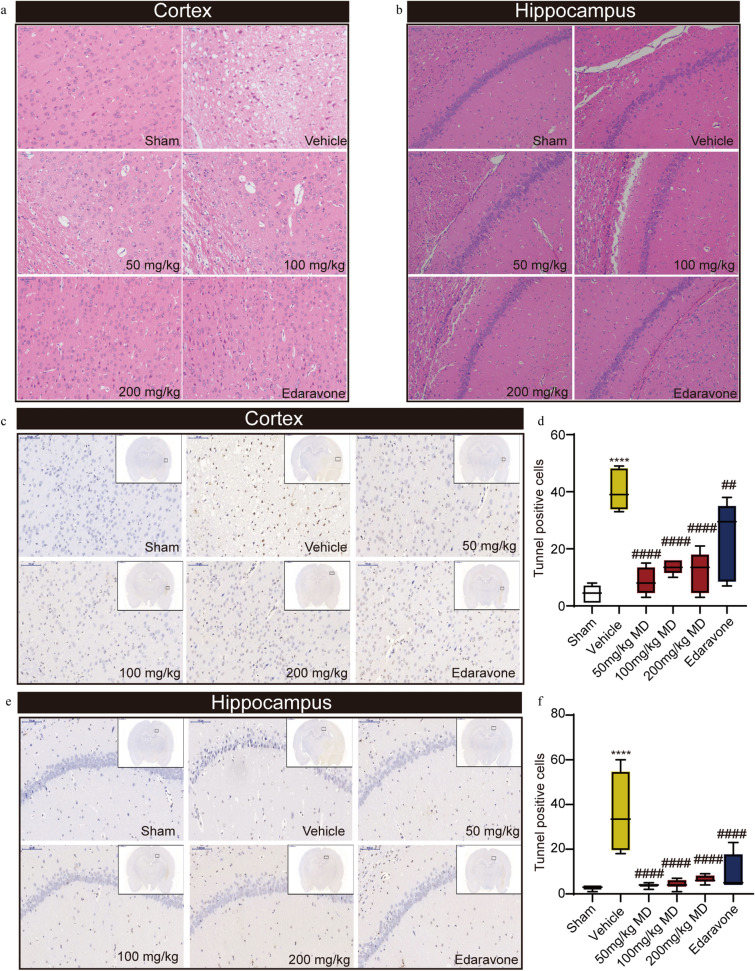
Meldonium attenuates brain injury and apoptosis in MCAO rats.** a**,** b** Meldonium (50, 100, or 200 mg/kg) or edaravone treatment attenuated the histopathology of the rat cerebral cortex and hippocampus; scale bar, 50 μm. **c**–**f** Meldonium (50, 100, or 200 mg/kg) or edaravone reduced neuronal apoptosis of the cerebral cortex and hippocampus; values are expressed as mean ± SD (n = 6). ****p < 0.0001 *vs*. the sham group; ^####^*p* < 0.0001 *vs*. the vehicle group; scale bar, 100 μm

Further, we found the neurons in cerebral cortex and hippocampus of the MCAO rats exhibited significant loose and sparse arrangements compared to the sham-operated rats (Fig. 2a, b). These changes were accompanied by karyolysis and pyknosis. However, all doses of meldonium and edaravone reduced the pathological changes caused by MCAO. Moreover, TUNEL staining demonstrated a significant increase in the number of brown apoptotic bodies in the cerebral cortex and hippocampus of MCAO rats compared to the sham-operated rats, indicating that neuronal apoptosis was induced by MCAO (Fig. 2c–f). Meldonium (50, 100 and 200 mg/kg) significantly reduced neuronal apoptosis in both the cortex (Fig. 2c, d) and the hippocampus (Fig. 2e, f).

These results indicate that meldonium significantly reduces the size of cerebral infarction, improves neurological function, enhances the motor abilities of MCAO rats, and maintains the morphology and orderly arrangement of neurons in the cerebral cortex and hippocampus.

### Meldonium improves mitochondrial morphology and function in MCAO rats

**Fig. 3:**
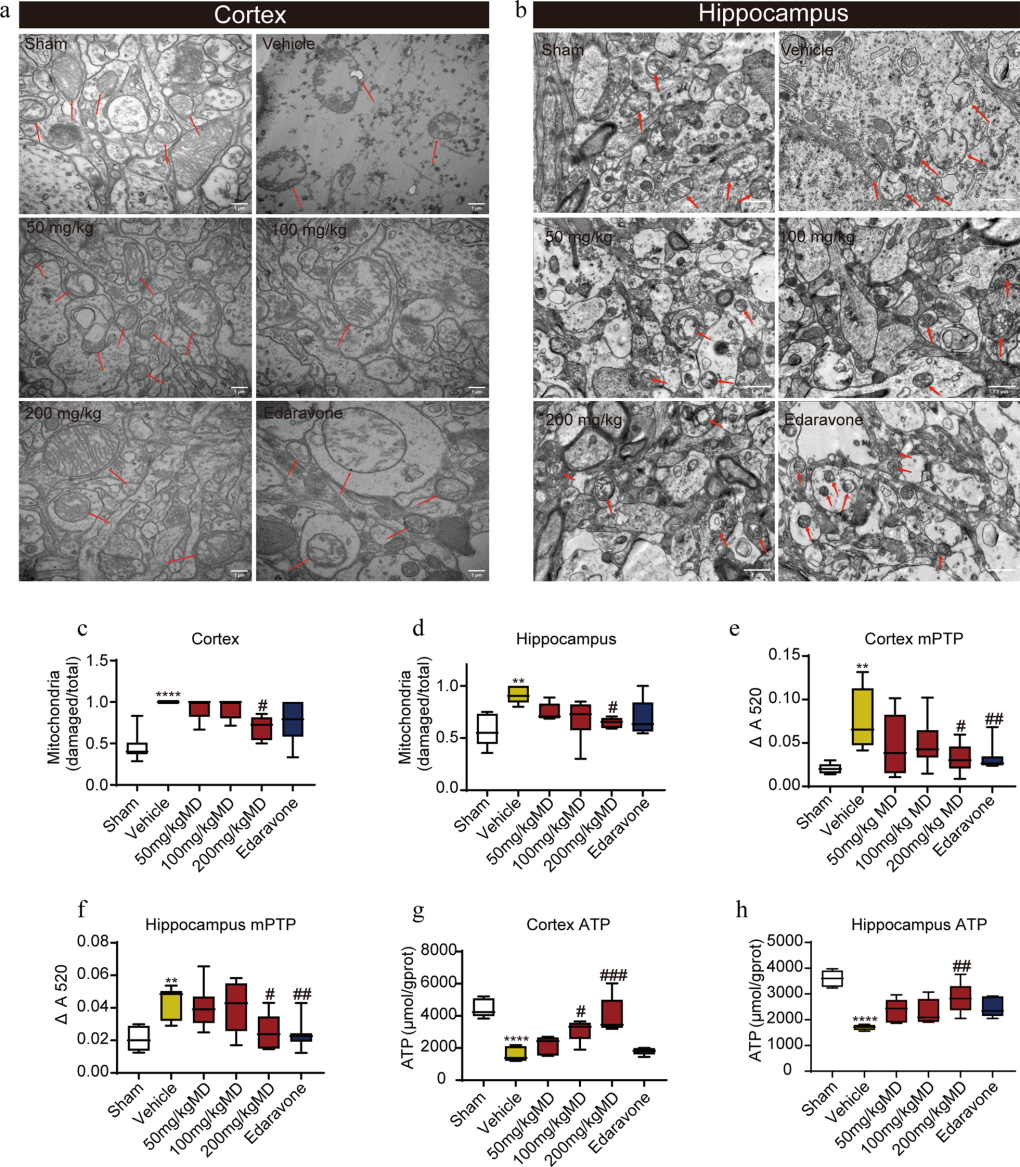
Meldonium treatment attenuates mitochondrial dysfunction induced by MCAO in the cerebral cortex and hippocampus. **a**–**d** TEM images of meldonium (50, 100, or 200 mg/kg) or edaravone treatment improved mitochondrial morphology in the cerebral cortex (40,000 magnifications) and hippocampus (4000 magnifications); scale bar, 1 μm; mitochondria were indicated by the red arrows; Values are expressed as mean ± SD (n = 6). ****p < 0.0001, **p＜0.01, *vs*. sham group; ^#^*p* < 0.05, *vs*. the vehicle group. **e**, **f** Meldonium inhibited the opening of the mPTP of the cortex (**e**) and hippocampus (**f**) at 24 h after reperfusion. Values are expressed as mean ± SD (n ≥ 8). **p < 0.01 *vs*. the sham group; ^#^*p* < 0.05, ^##^*p* < 0.01, *vs*. the vehicle group. **g**, **h** Meldonium increased ATP levels in the cortex (**g**) and hippocampus (**h**) at 24 h after reperfusion. Values are expressed as mean ± SD (n ≥ 8). ****p < 0.0001, *vs*. sham group; ^#^*p* < 0.05, ^##^*p* < 0.01, ^###^*p* < 0.001, *vs*. the vehicle group

In our previous study, target proteins of meldonium were investigated using the HuProt^TM^ human proteome microarray. KEGG pathway enrichment analysis indicated that the candidate meldonium target proteins are primarily involved in glycolysis and metabolic pathways [27]. To further understand the protective effect of medonium on mitochondria, the ultrastructure of neurons in the cerebral cortex and hippocampus was observed using transmission electron microscopy (TEM). The TEM results illustrate that, compared to the sham group, the morphology of neuronal mitochondria in the cortex and hippocampus of MCAO rats were significantly damaged, which was characterized by swollen mitochondria with decreased ridge density, and ruptured membranes (Fig. 3a–d). Treatment with meldonium improved the morphology of neuronal mitochondria.

To assess the effects of meldonium on mitochondrial function, we examined mPTP opening and ATP production in the cerebral cortex and hippocampus. Compared to the sham group, mPTP opening was significantly increased in both the cerebral cortex (Fig. 3e) and hippocampus (Fig. 3f) of MCAO rats. However, treatment with 200 mg/kg meldonium and edaravone effectively suppressed excessive mPTP opening in the cerebral cortex (Fig. 3e) and hippocampus (Fig. 3f) induced by MCAO. Additionally, ATP content was significantly lower in the cerebral cortex (Fig. 3g) and hippocampus (Fig. 3h) of MCAO rats compared to the sham group. However, treatment with 200 mg/kg meldonium significantly improved ATP content in the cerebral cortex (Fig. 3g) and hippocampus (Fig. 3h), whereas edaravone exhibited no significant protective effect on ATP content in the cerebral cortex and hippocampus of MCAO rats. Therefore, our findings indicated that meldonium protects mitochondrial morphology and function in the cerebral cortex and hippocampus of MCAO rats.

### Meldonium improves cerebral mitochondrial antioxidant stress capacity in MCAO rats

**Fig. 4:**
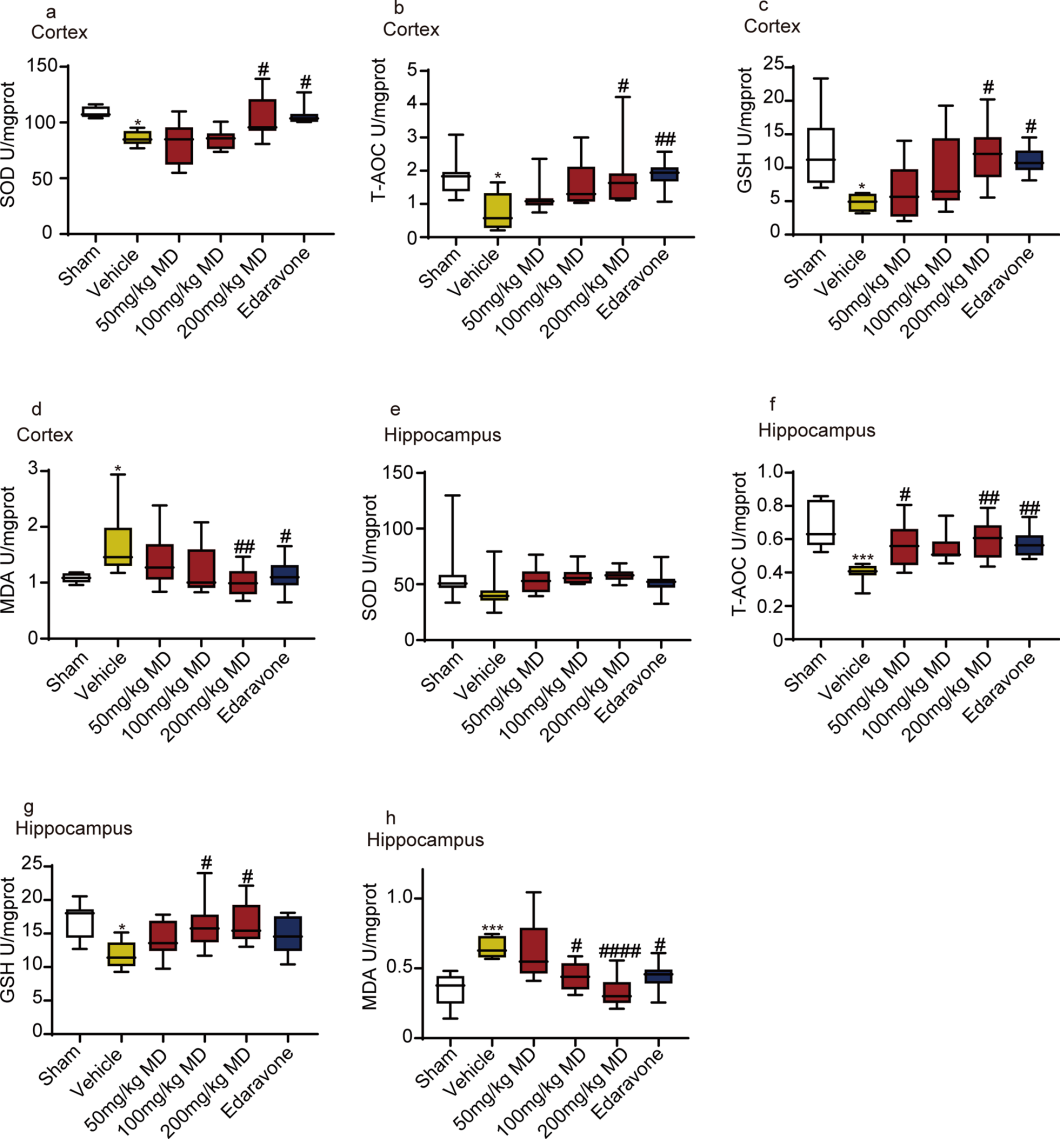
Meldonium improves antioxidant stress capacity of the cerebral cortex and hippocampus in MCAO rats. **a–h** Effects of mildronate on antioxidant indices in the cortex (**a**–**d**) and hippocampus (**e**–**h**) at 24 h after reperfusion: SOD activity, T-AOC activity, GSH, and MDA level. Values are expressed as mean ± SD (n ≥ 8). *p < 0.05, ***p < 0.01 *vs*. the sham group; ^#^*p* < 0.05, ^##^*p* < 0.01, ^####^*p* < 0.01 *vs*. the vehicle group

The antioxidant capacity of the mitochondria in the cortex and hippocampus of MCAO rats was investigated. The results demonstrated a significant decrease in SOD activity in the cerebral cortex of MCAO (Fig. 4a). T-AOC activity and GSH content were significantly decreased both in the cerebral cortex (Fig. 4b, c) and hippocampus (Fig. 4f, g) compared to the sham group. In contrast, the content of malondialdehyde (MDA) significantly increased in the cerebral cortex (Fig. 4d) and hippocampus (Fig. 4h), indicating MCAO injury decreased antioxidant capacity. In comparison with the MCAO group, both meldonium and edaravone significantly improved SOD activity, T-AOC activity, and GSH content in the cortex (Fig. 4a–c). Similarly, both drugs increased T-AOC activity and GSH content in the hippocampus (Fig. 4f, g). Furthermore, both meldonium and edaravone decreased MDA content in the cortex (Fig. 4d) and hippocampus (Fig. 4h). In conclusion, meldonium and edaravone improved the antioxidant capacity by reducing oxidative stress in the cortex and hippocampus of MCAO rats.

### Meldonium maintains neuronal mitochondrial function in OGD/R injury

**Fig. 5:**
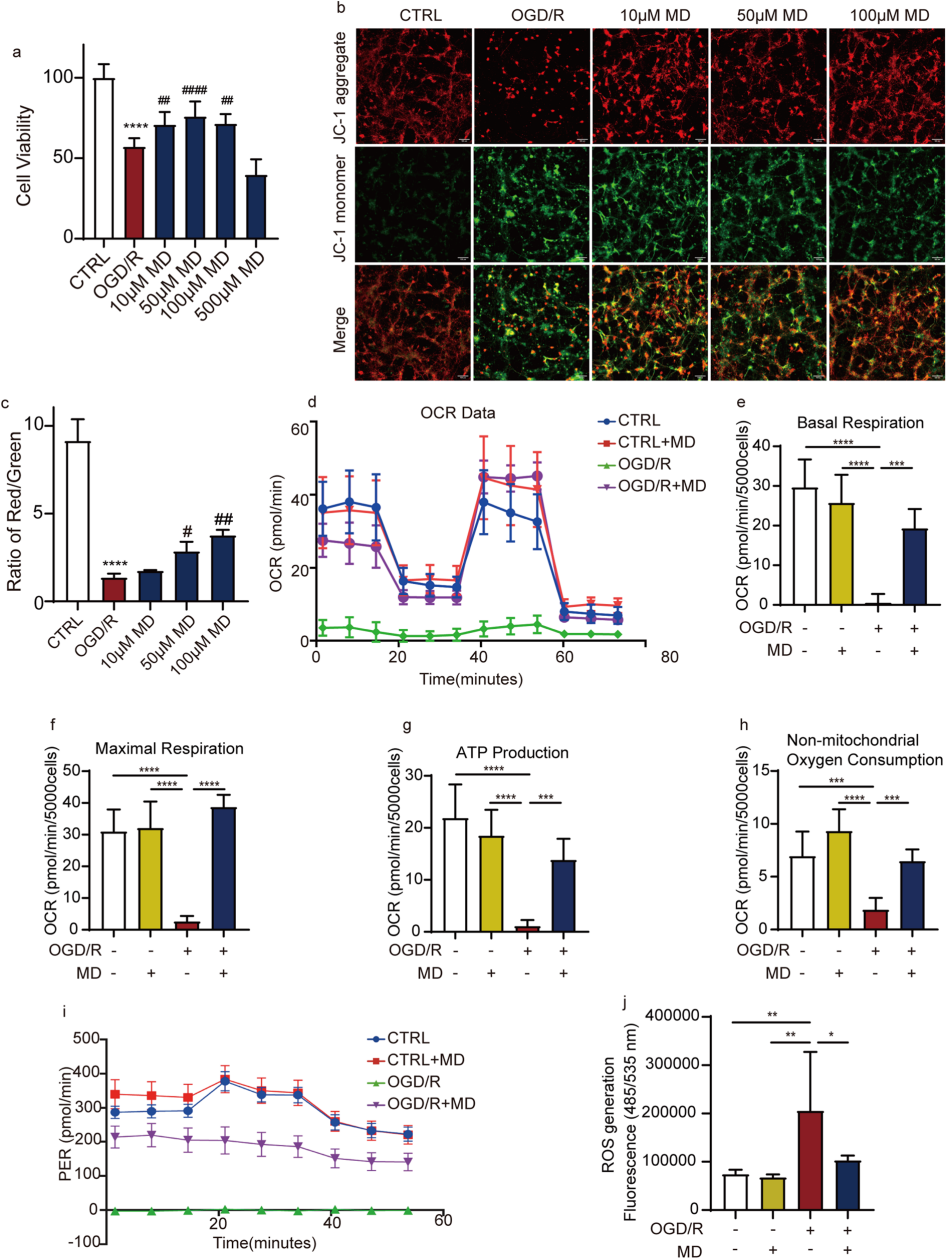
Meldonium treatment attenuates OGD/R-induced neuronal mitochondria damage in hippocampal neurons. **a** 10, 50 and 100 μM meldonium improves hippocampal neuronal viability. Values are expressed as the mean ± SD (n = 3). ****p < 0.0001, *vs*. CTRL group; ^##^*p* < 0.01, ^####^*p* < 0.0001 *vs*. the OGD/R group. **b**, **c** 50 and 100 μM meldonium improves mitochondrial transmembrane electric potential in hippocampal neurons. Images were assessed by high content screening (red, JC-1 aggregates; green, JC-1 monomers); values are expressed as mean ± SD (n = 3). ****p < 0.0001, *vs*. CTRL group; ^#^*p* < 0.05, ^##^*p* < 0.01*vs*. OGD/R group; scale bar, 100 μm. **d**–**i** The Seahorse XF Cell Mito Stress Test Kit and the Seahorse XF Real-Time ATP Rate Assay Kit were used to test the OCR (d-h) and ATP (i) formation rate diagram in hippocampal neurons, respectively. They were all detected by the Seahorse XF HS Mini Analyzer and analyzed using the XF HS Mini Control Software. Values are expressed as the mean ± SD (n = 4–6). ****p < 0.0001, ***p < 0.001, *vs*. OGD/R. (**j**) 100 μM meldonium inhibited the OGD/R injury induced elevations of ROS. Values are expressed as mean ± SD (n = 4–6). *p < 0.05, **p < 0.01, *vs*. the OGD/R group

To further investigate the effects of meldonium on neuronal mitochondria, we conducted a study using primary hippocampal neurons from embryonic day 18 fetal SD rats and created an in vitro model of OGD/R. The results from the MTT assay indicated that meldonium (10 μM, 50 μM, and 100 μM) significantly enhanced neuronal viability under OGD/R condition (Fig. 5a). Additionally, the JC-1 assay revealed an increase in green fluorescent J-monomer in the OGD/R group, indicating a substantial disruption of mitochondrial membrane potential (∆ψm). However, meldonium (50 μM and 100 μM) significantly preserved ∆ψm in neurons after OGD/R (Fig. 5b, c). Consequently, we employed 100 μM meldonium to further investigate its protective effect on hippocampal neurons.

We investigated the effect of meldonium on neuronal mitochondrial respiratory function and ATP production using mitochondrial stress and ATP real-time rate tests. The results revealed a significant decrease in OCR values in the OGD/R group compared to the control group (Fig. 5d), OGD/R injury significantly disrupted basal respiration, maximal respiration, ATP production and non-mitochondria oxygen consumption of mitochondria in hippocampal neurons (Fig. 5e–h). However, 100 μM meldonium protected these mitochondrial functions during OGD/R injury (Fig. 5e–h). PER values in the OGD/R group were also decreased, while treatment with meldonium could increase the PER values compared to the OGD/R group (Fig. 5i). ROS levels were also increased after OGD/R injury, which indicates that neurons were losing the capacity against oxidative stress, while meldonium significantly decreased the elevations of ROS (Fig. 5j). Consequently, we observed that 100 μM meldonium effectively preserved ∆ψm, protected mitochondrial respiratory function and ATP production, and inhibited excessive ROS release during OGD/R.

### Meldonium inhibits OGD/R induced mitochondrial excessive fission

**Fig. 6:**
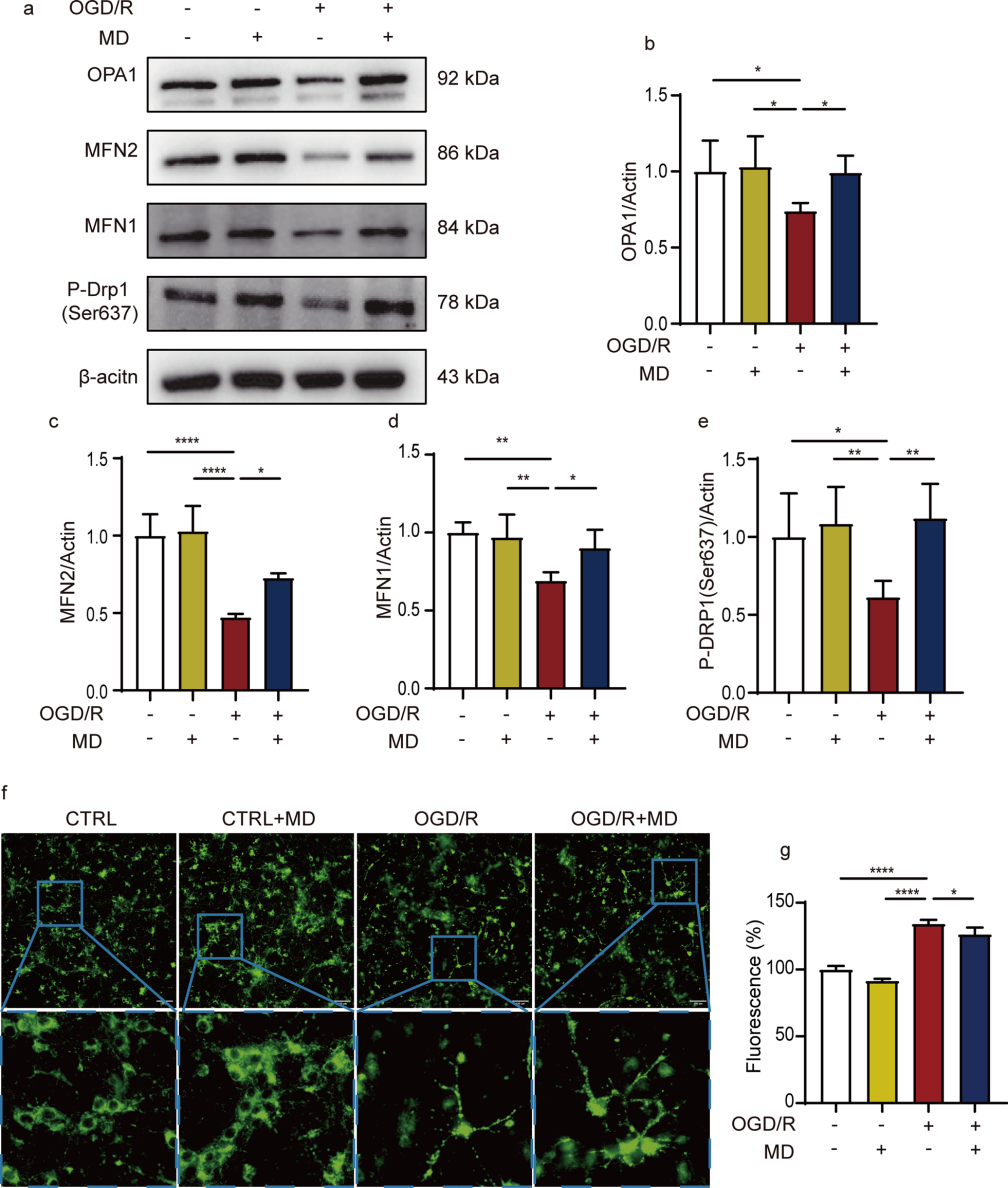
Meldonium attenuates OGD/R-induced imbalance in mitochondrial fission and fusion in hippocampal neurons. **a**–**e** Representative western blots and quantitative analysis of OPA1, MFN1, MFN2, and P-Drp1 (Ser637) with or without meldonium in cultured hippocampal neurons of the indicated groups in vitro. Values are expressed as mean ± SD (n = 3–5). *p < 0.05, **p < 0.01, ****p < 0.0001, *vs*. the OGD/R group;** f**,** g** Hippocampal neurons incubated with MitoTracker^®^ probes to label mitochondria and imaged by high-content screening. Values are expressed as the mean ± SD (n = 4). ****p < 0.0001, *p < 0.05, *vs*. OGD/R group

Mitochondria are highly dynamic organelles, and an imbalance in mitochondrial fission and fusion can lead to a loss of mitochondrial morphology and function, ultimately resulting in cell death. As shown in Fig. 6a–d, the expression of the mitochondrial fusion proteins MFN1, MFN2, and OPA1 were significantly decreased in hippocampal neurons after OGD/R injury compared to the control group (Fig. 6a–d). Additionally, the phosphorylation level of the fission protein Drp1 at serine 637 (p-Drp1S637), a site that inhibits fission, was significantly lower than that in the control group (Fig. 6a, e). These findings indicated a significant disruption in mitochondrial dynamics in hippocampal neurons after OGD/R injury. In contrast, treatment with meldonium significantly increased the protein expression of mitochondrial fusion proteins and p-Drp1S637 during OGD/R compared to the OGD/R group (Fig. 6a–e). Besides, Neuronal mitochondria lost their normal morphology during OGD/R and showed numerous punctured fragments (Fig. 6f, g), possibly related to excessive fission and damaged fusion processes. Meldonium treatment ameliorated the disruption of neuronal mitochondrial morphology during OGD/R (Fig. 6f, g). These results showed that meldonium balanced mitochondrial fission and fusion, and improved mitochondrial morphology during OGD/R.

### Meldonium inhibits mitochondria-dependent apoptosis induced by excessive openness of mPTP through the Akt/GSK-3β signaling pathway

**Fig. 7:**
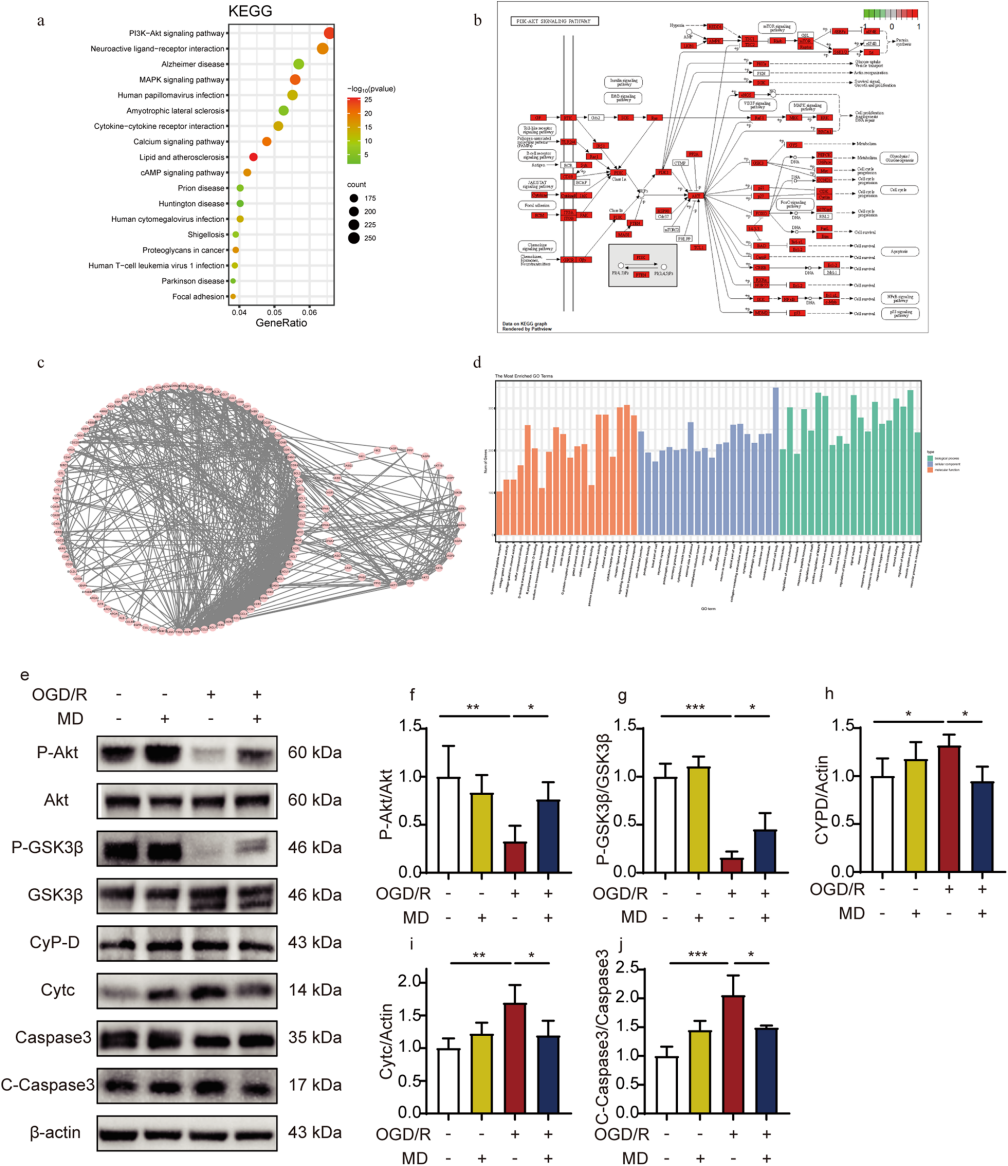
Meldonium inhibits hippocampal neuronal apoptosis through the Akt/GSK3β signaling pathway in vitro. **a** Bubble chart of stroke-related genes KEGG pathway enrichment analysis. The rows represent the ratio, and the columns represent the KEGG pathway. **b** PI3K-Akt pathway-related genes. The red-highlighted areas denote genes associated with stroke development. **c** Stroke-related genes’ PPI networks. **d** GO enrichment analysis of stroke-related genes. The abscissa indicates the enriched GO, and the ordinate is the number and ratio of genes.** e**–**j** Representative western blots and quantitative analysis of P-Akt, Akt, P-GSK3β, GSK3β, CyPD, Cytc, Caspase3, and C-Caspase3 with or without 100 μM meldonium in cultured neurons from the indicated groups. Values are expressed as mean ± SD (n = 3–4); *p < 0.05, **p < 0.01, ***p < 0.001, *vs*. the OGD/R group

To investigate the mechanism underlying the meldonium-induced improvement in mitochondrial function, we analyzed 6980 stroke-related genes sourced from various databases. Cluster analysis revealed these genes were strongly correlated with the PI3K-Akt signaling pathway (Fig. 7a, b). Furthermore, gene ontology analysis revealed the involvement of these stroke-related genes in several biological processes, including responses to oxygen levels and neuronal death (Fig. 7c). The PPI networks of these genes, based on the Search Tool for the Retrieval of Interacting Genes/Proteins (STRING) database, indicated several functional groups, such as Akt and caspase3 (Fig. 7d), which were potentially significant in stroke pathology.

**Fig. 8:**
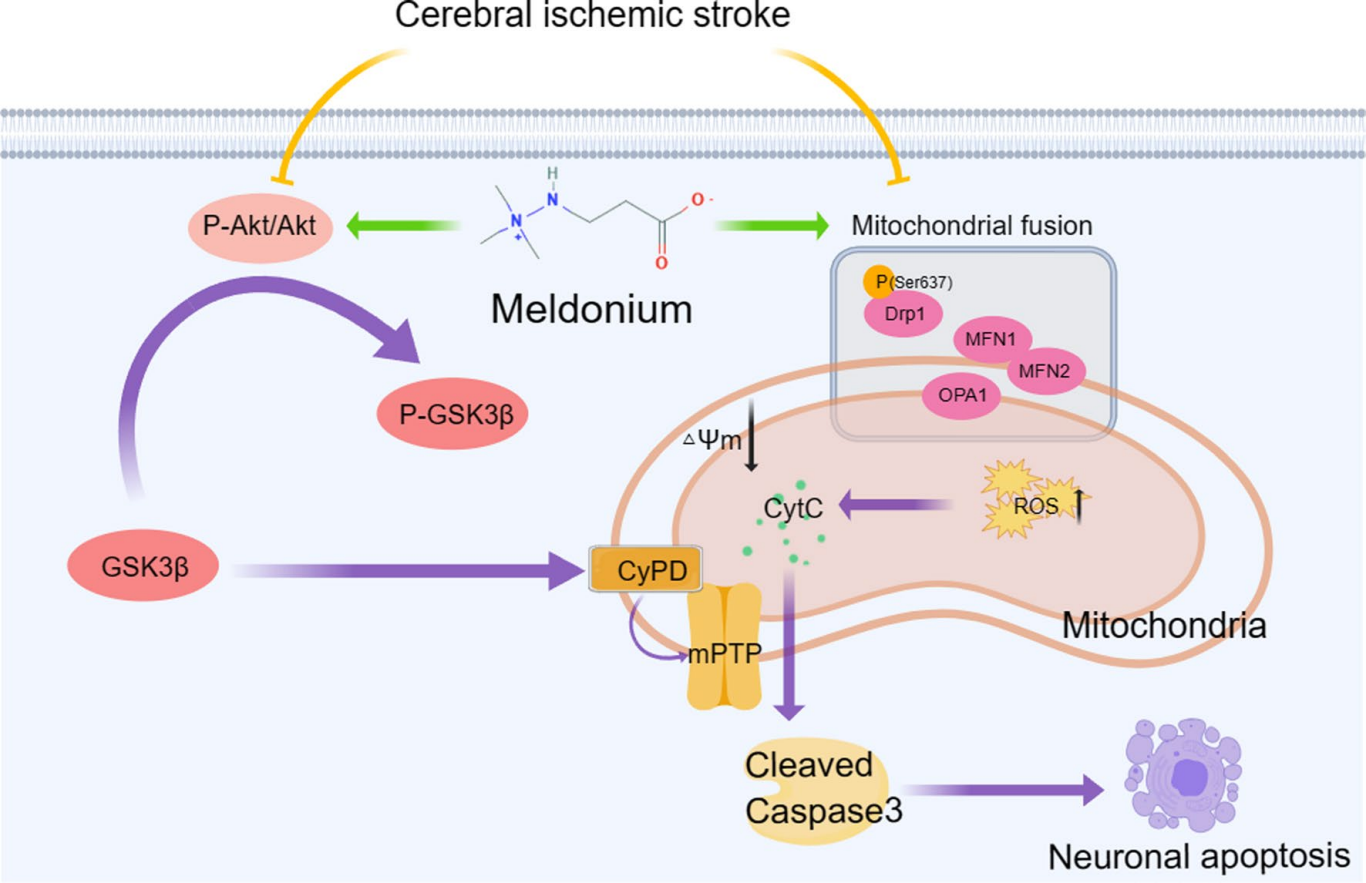
Meldonium protects neurons against CIRI by maintaining mitochondrial morphology and function. Meldonium serves a dual role: it maintains mitochondrial dynamics by protecting the mitochondrial fusion process. Additionally, it decreases the cellular ROS levels and protects △Ψm of neuronal mitochondria. Furthermore, it activates the AKT/GSK3β/CyPD pathway to restrain the over-openness of mPTP during CIRI, which further inhibits the release of Cyt C from mitochondria into the cytoplasm and caspase3-mediated neuronal apoptosis

Numerous studies have indicated that the Akt/GSK-3β signaling pathway is crucial in regulating the initial mPTP opening and is essential in the mitochondria-dependent apoptosis pathway. The western blot results demonstrated that the phosphorylation levels of Akt and GSK-3β in neurons were significantly decreased after OGD/R compared with the control group (Fig. 7e–j)). Conversely, the expression levels of CyPD, Cyt C, and the cleaved forms of Caspase3 (C-Caspase3) significantly increased (Fig. 7e–j). Additionally, meldonium treatment significantly increased the phosphorylation levels of Akt and GSK-3β while significantly inhibiting Cyt C release and caspase-3 activation (Fig. 7e–j). These findings indicated that meldonium could potentially prevent excessive mPTP opening by regulating the Akt/GSK-3β signaling pathway, thereby inhibiting the mitochondria-dependent apoptosis pathway and ultimately protecting neuronal survival (Fig. 8).

## Discussion

Cardio-cerebrovascular diseases remain the main killer worldwide [42–46]. Among which, ischemic stroke is a cerebrovascular disease caused by arterial occlusion that triggers multiple biochemical events [47, 48], including neuronal excitotoxicity, oxidative stress, inflammation, and apoptosis. These events lead to cell death and brain infarction [49–51]. They can lead to various neurological deficits, such as paralysis, speech difficulties, cognitive impairments, dementia, or even death [52, 53]. The current clinical therapeutic treatments for stroke include thrombolysis, treatment of stroke-related comorbidities, and prevention of recurrence [54]. However, these treatments are limited in their ability to promote neuronal regeneration and restore brain function. Furthermore, the main challenge in repairing nerve function damage after stroke is posed by the postmitotic neurons with minimal regenerative capacity [55]. Researchers and clinicians have recently explored various strategies to protect cell survival, enhance post-stroke neuronal regeneration, and improve functional recovery. Thrombolytics and neuroprotective agents are the two main categories of drug candidates based on their mechanisms of action [56]. Neuroprotective agents are drugs that halt the ischemic cascade, prevent secondary injury, or decrease the loss of vulnerable neurons in the ischemic penumbra [56]. These agents employ various mechanisms, such as antioxidants, neuron stimulants, calcium channel antagonists, and free radical scavengers, to protect against stroke-related injury [56]. While most neuroprotective agents have shown promise in animal models, translating them into clinical trials remains a substantial problem for researchers and developers. Meldonium, a leading cardiovascular agent particularly used for angina and heart failure, has undergone extensive study for its potential protective mechanism against ischemic injury and improving cognitive function [57]. It preserves cardiac sarcoplasmic reticulum Ca^2+^-ATPase and hexokinase type I during myocardial infarction, exhibiting cardioprotective activity effects by activating NO synthetase, which stimulates the synthesis of GBB [25, 58]. Additionally, meldonium possesses antioxidant properties that can alleviate oxidative stress in the heart [13]. Several studies have indicated that meldonium’s heart-protective mechanisms are linked to glucose metabolism facilitation, thereby maintaining mitochondrial function [16, 59, 60]. Additionally, numerous studies have highlighted meldonium’s significant role in various CNS diseases. Meldonium regulates the expression of nerve and glial cell proteins in rats with Parkinson's disease and reduces hippocampal amyloid deposition in mice with Alzheimer's disease [21, 61]. It can also ameliorate the pathological brain tissue injury in zidovudine-induced neurotoxic model mice, regulate the expression of learning- and memory-related proteins in two classical amnesia (stress and haloperidol models) rat models [62], and improve nerve function in an endothelin-1-induced ischemic stroke rats model [33]. Additionally, Zhu et al. conducted an extensive multicenter clinical trial demonstrating the safety and efficacy of meldonium for the clinical treatment of stroke patients [22]. These investigations showed meldonium's potential effectiveness in treating CNS illnesses, but they neglected its neuroprotective effect and failed to thoroughly study the mechanism underlying its neural protection. According to our study, we proved that meldonium could improve neurological function, considerably lessen pathological neuronal injury in the cortex and hippocampus, and reduce infarct volume in MCAO rats. Reportedly, over 40% of patients with stroke experience persistent motor dysfunction [63]. And in our study, the retention time in MCAO rats was shown to be greatly increased by meldonium, suggesting that the drug may have a positive impact on motor function.

Additionally, we investigated the neuroprotective mechanism of meldonium against CIRI. In our previous studies, the candidate meldonium target proteins were primarily associated with glycolysis and metabolic pathways [27]. This indicated that meldonium might have a potential neuroprotective effect against CIRI by improving mitochondrial function. Moreover, Increasing evidence reveals that protecting mitochondrial function is essential for improving neuronal survival and function after CIRI [64]. And it is also reported that electrical activity, neurotransmitter synthesis and release, nerve excitation and conduction, and other processes in brain cells require a significant amount of energy, mostly from glucose consumption [65]. During a stroke, the brain is deprived of glucose and oxygen [66], preventing the mitochondria from supplying sufficient energy for neuronal survival. This disrupted energy balance induces mitochondria to produce excessive ROS, oxygen-free radicals, and an imbalance of the endogenous antioxidant system, leading to increased lipid peroxidation and neuronal apoptosis [67]. A higher content of MDA, a product of lipid peroxidation, indirectly indicates the severity of oxygen free radicals (OFR) attacks on cells [68]. SOD is an essential enzyme in the endogenous antioxidant system, reflecting its ability to eliminate OFR [68]. GSH is living cells’ primary non-enzymatic reducing agent, functioning as a free radical scavenger that inhibits lipid peroxidation [68]. T-AOC is an indicator that measures the cumulative impact of various antioxidant substances and enzymes, representing the most direct measure of antioxidant capacity [68]. In addition, our study indicated that meldonium significantly improved the antioxidant capacity of the cortex and hippocampus in MCAO rats. And during CIRI, oxidative stress and ATP depletion can induce extensive mPTP opening, leading to cytochrome c release and the activation of caspase cascades, culminating in cell apoptosis [69]. Therefore, the mPTP is often described as the cell's life-and-death switch. Consequently, our study highlights that meldonium increases the overall ATP produced in the cerebral cortex and hippocampus of MCAO rats and inhibits excessive mPTP opening, indicating that meldonium may protect neuronal survival by inhibiting mitochondria-dependent apoptosis and maintaining basic mitochondrial function.

We further investigated that the protective effect of meldonium on mitochondria using a rat primary hippocampal neuronal OGD/R model. Depolarization of the ∆ψm is a crucial event in the early stage of apoptosis, which can impede the ETC process and induce mPTP opening to trigger apoptosis [70]. Ischemia-induced neuronal apoptosis has been widely investigated and confirmed [69]. Our results also demonstrated that meldonium inhibited neuronal apoptosis induced by MCAO. And meldonium effectively maintained ∆ψm under these OGD/R injurious conditions. Additionally, we found that meldonium had a significant protective effect on mitochondrial dynamic-related proteins during OGD/R injury. Meanwhile, mitochondrial function is maintained by a balance between fission and fusion, known as “mitochondrial dynamics”. The mitochondrial fission process involves the enrichment, contraction, and fragmentation of dynamin-related protein 1 (DRP1). Reportedly, Drp1 phosphorylation at serine 637 can inhibit mitochondrial fission and protect against the mitochondrial fusion process [71]. The mitochondrial fusion process depends on mitofusin 1/2 (MFN1/2) and optic atrophy 1 (OPA1), expressing in the outer and inner membranes, respectively [67, 72]. And defective mitochondrial fusion is an early event in ischemic stroke, resulting in the inability of mitochondria to reacquire the respiratory chain and mitochondrial DNA [73]. KW et al. found that early-onset stroke might be linked to a novel MFN2 mutation [74]. And in our study, meldonium significantly increased the expression of MFN1/2, OPA1, and p-Drp1S637 proteins and improved the mitochondrial morphology of neurons during OGD/R, thereby reducing excessive fission-induced mitochondrial fragmentation. Additionally, we comprehensively investigated basic, maximal and ATP-linked respiration, and non-mitochondrial oxygen consumption in neuronal mitochondria using the mitochondrial stress test. And also, we simultaneously quantified mitochondrial ATP levels in living cells using an ATP real-time rate test. Therefore, our findings indicated that meldonium treatment significantly improved the OCR and PER of neurons with OGD/R injury. All in all, these findings indicated that meldonium effectively improved mitochondrial respiratory function and restored energy supply, thereby maintaining the primary function of neuronal mitochondria during OGD/R injury.

Additionally, cerebral ischemia induces several deleterious effects, such as the reduction or depletion of ATP production, increased generation of free radicals, massive excitatory amino acid release, disrupted calcium homeostasis, NO production, and upregulated production of inflammatory cytokine cascades, leading to an intracellular environment disorder [50, 75]. These injury signals would upregulate mitochondria-dependent neuronal apoptosis-related genes and proteins through the apoptotic signal transduction pathway, thereby triggering the execution stage of mitochondria-dependent apoptosis. Our experiment demonstrated that MCAO resulted in the excessive opening of the mPTP and the release of cytochrome c, a critical step in the apoptotic pathway [76]. Once Cyt C is released from the mitochondrial inner membrane into the cytoplasm, it combines with the apoptotic protease activating factor 1. This complex activates caspase-9, subsequently activating caspase-3 to initiate the caspase cascade, ultimately leading to apoptosis [77]. Akt directly affects the mitochondria, leading to either protection against oxidants or mPTP opening [78]. Phosphorylation of Akt consequently inhibits the activity of GSK3β by phosphorylating its Ser-9 residue. Inactivated GSK3β reduces the expression of CyPD in mitochondria, directly inhibiting the opening of mPTP [79]. Consequently, the activation of Akt/GSK3β can reduce the release of Cyt C into the cytoplasm and ultimately inhibit cell apoptosis [78]. We screened 6980 stroke-related genes from six databases, and their pathways enriched by the KEGG analysis were highly related to PI3K-Akt. Similarly, our study found that meldonium significantly affects the expression of proteins related to the mitochondria-dependent apoptosis pathway by activating Akt/GSK-3β, thereby inhibiting and reducing the excessive release of Cyt C into the cytoplasm, therefore preventing the activation of caspase3 and ultimately inhibiting neuronal apoptosis (Fig. 8).

In conclusion, our study found that meldonium promoted neuronal survival by improving the function and antioxidant ability of mitochondria during CIRI, thereby significantly reducing infarct size and improving motor ability in MCAO rats. Mechanistically, meldonium restrained mitochondrial excessive fission and loss of ∆ψm to maintain its morphology and respiratory function. Furthermore, meldonium activated the AKT/GSK3β/CyPD pathway to inhibit mitochondria-dependent neuronal apoptosis. However, this study had a few limitations. For example, besides improving neuronal survival during acute injury, recovery of neural function should also be considered. Further research is required to investigate whether meldonium can effectively restore neuronal axonal growth, dendritic remodeling, and the formation of a new neural network after acute brain injury. This research provides comprehensive basic data for meldonium studies in CNS diseases.

## Data Availability

Not applicable.

## Abbreviations

CIRI: Cerebral ischemia/reperfusion injury
tPA: Tissue plasminogen activator
FDA: Food and Drug Administration
ROS: Reactive oxygen species
ACI: Acute cerebral infarction
iNOS: Inducible nitric oxide synthase
CNS: Central nervous system
MCAO: Middle cerebral artery occlusion
DMEM: Ulbecco's Modified Eagle Medium
OGD/R: Oxygen–glucose deprivation reperfusion
PER: Proton efflux rate
OCR: Oxygen consumption rate
CCA: Common carotid artery
TTC: 2,3,5-Triphenyltetrazolium chloride
HE: Hematoxylin and Eosin
mPTP: Mitochondrial permeability transition pore
PPI: Protein–protein interaction
KEGG: Kyoto Encyclopedia of Genes and Genomes
GO: Gene Ontology
NDS: Neurological deficit score
SOD: Superoxide dismutase
T-AOC: Total antioxidant capacity
GSH: Glutathione
ATP: Adenosine triphosphate
Rot/AA: Rotenone/antimycin A
CyP–D: Cyclophilin D
Cyt C: Cytochrome C
OPA1: Optic atrophy 1
DRP1: Dynamin-related protein 1
TTD: Therapeutic Target Database
CTD: Comparative Toxicogenomics Database
∆ψm: Mitochondrial membrane potential
